# Feasibility, acceptability, and tolerability of operation calm: a mixed-methods evaluation with children, parents, and clinicians

**DOI:** 10.3389/frhs.2026.1912318

**Published:** 2026-10-28

**Authors:** Brandon Benchimol-Elkaim, Bassam Khoury, Argerie Tsimicalis

**Affiliations:** 1 Department of Educational and Counselling Psychology, Faculty of Education, McGill University, Montreal, QC, Canada; 2 Ingram School of Nursing, Faculty of Medicine and Health Sciences, McGill University, Montreal, QC, Canada

**Keywords:** feasibility, implementation, mindfulness, mixed methods, nature-based intervention, pediatric procedural anxiety, virtual reality

## Abstract

**Background:**

Anxiety related to medical and dental procedures is common in childhood. Brief, portable interventions that combine immersive virtual reality (VR), mindfulness, and nature exposure may help children practice self-regulation, but the feasibility, acceptability, and tolerability of Operation Calm require evaluation before clinical efficacy testing.

**Methods:**

This protocol describes a community-based, single-arm, dynamic mixed-methods pre–post feasibility study. Approximately 50 children and adolescents aged 6–17 years, up to 50 parents or legal guardians, and 50 pediatric clinicians or supervised trainees will be recruited across Canada. Children will complete one approximately 10-minute self-guided Operation Calm session using a smartphone-compatible VR headset and one of five 360° nature scenes paired with standardized mindfulness narration. Parents and clinicians will review one scene in an approved web-based format. Primary domains are recruitment and procedural feasibility, acceptability, engagement, usability, appropriateness, perceived usefulness, implementation feasibility, and child tolerability. Children will complete the Children's Anxiety Meter–State and a current calmness rating immediately before and after VR, the Pediatric Simulator Sickness Questionnaire after VR, and a child evaluation. Parents and clinicians will complete group-specific evaluations with quantitative and open-ended items.

**Results:**

Feasibility and evaluation outcomes will be summarized descriptively with transparent denominators and confidence intervals where informative. Exploratory paired pre–post changes in child anxiety and calmness will be analyzed using paired-samples t tests or Wilcoxon signed-rank tests, with effect sizes and confidence intervals. Open-ended responses will undergo qualitative content analysis, and quantitative and qualitative findings will be integrated using a convergent mixed-methods approach. Scene-level profiles will be descriptive rather than confirmatory.

**Conclusions:**

This early-stage study will determine whether Operation Calm can be delivered safely and consistently outside hospital care and identify refinements needed before a clinical feasibility study or randomized trial. Immediate pre–post changes will be interpreted as exploratory and will not establish efficacy.

**Clinical Trial Registration:**

https://clinicaltrials.gov, ClinicalTrials.gov NCT07694934.

## 1 Introduction

Anxiety related to medical and dental procedures is common among children and adolescents and may involve fear of pain, needles, anesthesia, unfamiliar environments, bodily harm, separation from caregivers, uncertainty, and loss of control. Preoperative anxiety alone affects a substantial proportion of pediatric patients, with younger age, previous healthcare experiences, parental anxiety, temperament, and procedural characteristics among the factors that may influence its occurrence (1, 2). Heightened anxiety can increase distress and perceived pain, complicate cooperation with healthcare procedures, contribute to difficult anesthesia induction, and be associated with unfavourable postoperative behavioural outcomes (3, 4). These concerns arise not only during surgery but also during vaccination, venipuncture, dentistry, imaging, and other potentially stressful procedures. The repeated and developmentally varied nature of healthcare encounters underscores the value of supports that can be used before different procedures rather than only within a single hospital pathway.

Current non-pharmacological approaches include education, procedural familiarization, therapeutic play, child-life support, parental presence, music, games, audiovisual distraction, and relaxation strategies. Child-life specialists provide developmentally appropriate and emotion-focused support intended to strengthen coping and reduce distress during healthcare encounters, although the availability and use of these services vary across settings (5). Reviews of pediatric preoperative interventions similarly indicate that active and passive distraction strategies can reduce anxiety, although intervention content, delivery, and effects differ considerably across studies (6). Many technology-based interventions also rely primarily on distraction or familiarization. Although immediate distraction can reduce distress, it may provide limited opportunity to understand and practise a transferable coping skill. A brief intervention that is engaging, portable, developmentally appropriate, and usable across settings could therefore complement existing care without being represented as a replacement for clinical support.

### 1.1 Virtual reality, mindfulness, and virtual nature

VR uses a head-mounted display to create an immersive audiovisual experience and has increasingly been evaluated as a non-pharmacological tool for pediatric pain and anxiety. Across pediatric procedures, immersive VR can capture attention, reduce awareness of unpleasant stimuli, and improve engagement. Systematic reviews have reported promising effects on procedural pain and anxiety, although the evidence is heterogeneous across procedures, intervention formats, and outcomes (3, 7). An umbrella review also found consistent reductions in children's needle-related procedural pain, while noting substantial heterogeneity and overlap across the available meta-analyses (8).

VR has demonstrated preliminary feasibility, clinical utility, and tolerability in pediatric perioperative care, including delivery from the preoperative waiting area through anesthesia induction (9). However, that study evaluated an interactive VR distraction game rather than mindfulness-based virtual nature. Its findings therefore support the feasibility of perioperative VR delivery but do not establish the feasibility or effectiveness of Operation Calm's specific intervention components. Important gaps remain regarding intervention mechanisms, developmental suitability, safety, implementation, and longer-term outcomes (7). Hardware, content, exposure duration, supervision, and context may influence immersion, comfort, usability, accessibility, and cybersickness. These implementation features should therefore be evaluated alongside perceived benefit.

Mindfulness involves intentional, non-judgmental attention to present-moment experience (10). Practices such as paced breathing, sensory grounding, body awareness, and gentle redirection of attention may support emotion regulation by changing how individuals attend to and respond to emotional and physiological experiences (11). Meta-analytic evidence suggests that mindfulness-based interventions can reduce psychological distress in young people, although effects vary across outcomes, populations, and study designs (12). More broadly, a review of 44 meta-analyses found that mindfulness-based interventions generally performed better than passive control conditions, while effects were smaller and less consistently significant when compared with active interventions (13). These findings support continued investigation while cautioning against assuming that mindfulness is uniformly effective across populations and contexts.

Traditional audio-only or didactic mindfulness practice may be difficult for some children to understand or sustain. VR may provide a more concrete and structured attentional environment by reducing competing environmental distractions and directing attention toward present-moment sensory experience (14). Multisensory virtual environments may also strengthen the connection between bodily awareness, attention, and emotional experience, although these proposed mechanisms require careful empirical evaluation (15). Operation Calm therefore uses VR not solely as a means of distraction but as an environment in which children can actively practise breathing, grounding, and mindful sensory attention.

Natural environments have long been proposed to support restoration and psychological well-being through processes such as attentional recovery and an intrinsic human affinity for natural systems (16, 17). Early clinical evidence suggested that even indirect exposure to nature, such as a view through a hospital window, could influence recovery-related outcomes (18). More recent evidence associates exposure to natural environments with restoration, positive affect, reduced stress, and a range of physical and mental health outcomes (19, 20).

Immersive 360° nature videos make exposure to natural environments possible irrespective of location and may support positive affect, mood, and restoration (21, 22). Virtual nature is not assumed to reproduce all the benefits of direct contact with nature, but it may offer a practical alternative when access to natural settings is limited (22). Combining virtual nature with guided mindfulness may be complementary: nature scenes provide concrete sensory anchors, while VR may reduce environmental distraction and increase presence during mindfulness practice (14).

### 1.2 Theoretical framework and intervention development

Operation Calm was developed from a synthesis of evidence concerning pediatric perioperative anxiety, mindfulness, immersive VR, and exposure to virtual nature (23). That narrative review identified a theoretical rationale for combining these components while also highlighting the absence of direct empirical research evaluating an integrated nature-based VR mindfulness intervention for pediatric perioperative anxiety.

Operation Calm is further informed by the Embodied and Embedded Mindfulness and Compassion Framework (EEMCF), which conceptualizes mindful awareness as grounded in bodily experience and shaped through interaction with social and environmental contexts (24). Within Operation Calm, paced breathing and awareness of bodily sensations support embodied attention, while sensory engagement with virtual natural environments supports environmental embeddedness. Reflection on calm, safety, and relaxation provides a developmentally accessible bridge between the immediate virtual experience and possible future coping.

The intervention was originally developed for pediatric perioperative anxiety. Loss of the original clinical study site prompted a scientifically substantive redesign rather than a change in venue alone. The present study no longer tests clinical efficacy, randomizes pediatric surgical patients, compares Operation Calm with standard care, or includes perioperative follow-up. Instead, it addresses earlier-stage questions that should precede a larger clinical trial: Can intended users access and complete the intervention? Is it understandable, acceptable, and tolerable? Which content and delivery features require refinement? Do parents and clinicians view it as developmentally and clinically appropriate? A community sample is suited to these questions because diagnostic status and an imminent procedure are not required to evaluate the intervention's usability, acceptability, and immediate tolerability.

### 1.3 Perspectives of children, parents, and clinicians

Children provide direct evidence regarding engagement, comprehension, comfort, immersion, and perceived helpfulness. Parents influence access, setup, supervision, and eventual use and can assess developmental fit and family practicality. Pediatric clinicians can evaluate clinical relevance, workflow compatibility, safety, potential settings of use, implementation barriers, and required resources. This is particularly important because promising results under controlled research conditions do not necessarily establish that an intervention will be feasible within routine pediatric care. Integrating the perspectives of children, parents, and clinicians therefore supports user-centred refinement before a larger clinical feasibility study or randomized controlled trial.

### 1.4 Objectives and research questions

The primary objectives are to evaluate recruitment and procedural feasibility; characterize acceptability, engagement, usability, appropriateness, and perceived usefulness from the perspectives of children and parents; characterize clinical relevance and implementation feasibility from the perspective of clinicians; and assess child tolerability, including cybersickness, fatigue, discomfort, and early discontinuation. Secondary and exploratory objectives are to examine willingness to use Operation Calm before a future procedure, explore immediate pre–post changes in state anxiety and calmness, describe response profiles across five virtual nature scenes, and obtain recommendations regarding content, language, duration, delivery, accessibility, safety, and implementation.

The study asks whether eligible participants can be recruited and complete study procedures with acceptable levels of missing data and technical difficulty; whether children and parents find the intervention understandable, engaging, useful, and appropriate; whether clinicians find it relevant and feasible; what VR-related symptoms occur; whether children report immediate exploratory changes in anxiety and calmness; and where quantitative ratings and qualitative feedback converge or diverge. We anticipate high procedural completion, generally favourable acceptability, and mostly absent or mild transient VR symptoms. Any observed pre–post change will be treated as hypothesis-generating and will not be interpreted as evidence of clinical efficacy.

## 2 Method and analysis

### 2.1 Study design and reporting framework

This is a community-based, single-arm, dynamic mixed-methods pre–post feasibility study, as depicted in Figure 1. All children receive one Operation Calm session. Parents and clinicians review one scene and complete group-specific evaluations. Quantitative and qualitative data are collected within the same evaluation forms and integrated during interpretation. Dynamic feasibility refinements may clarify instructions or troubleshooting procedures; material changes to the intervention, eligibility criteria, outcomes, risks, or consent materials require prior Research Ethics Board (REB) approval. The protocol is numbered 26-08-078 (McGill REB).

**Figure 1:**
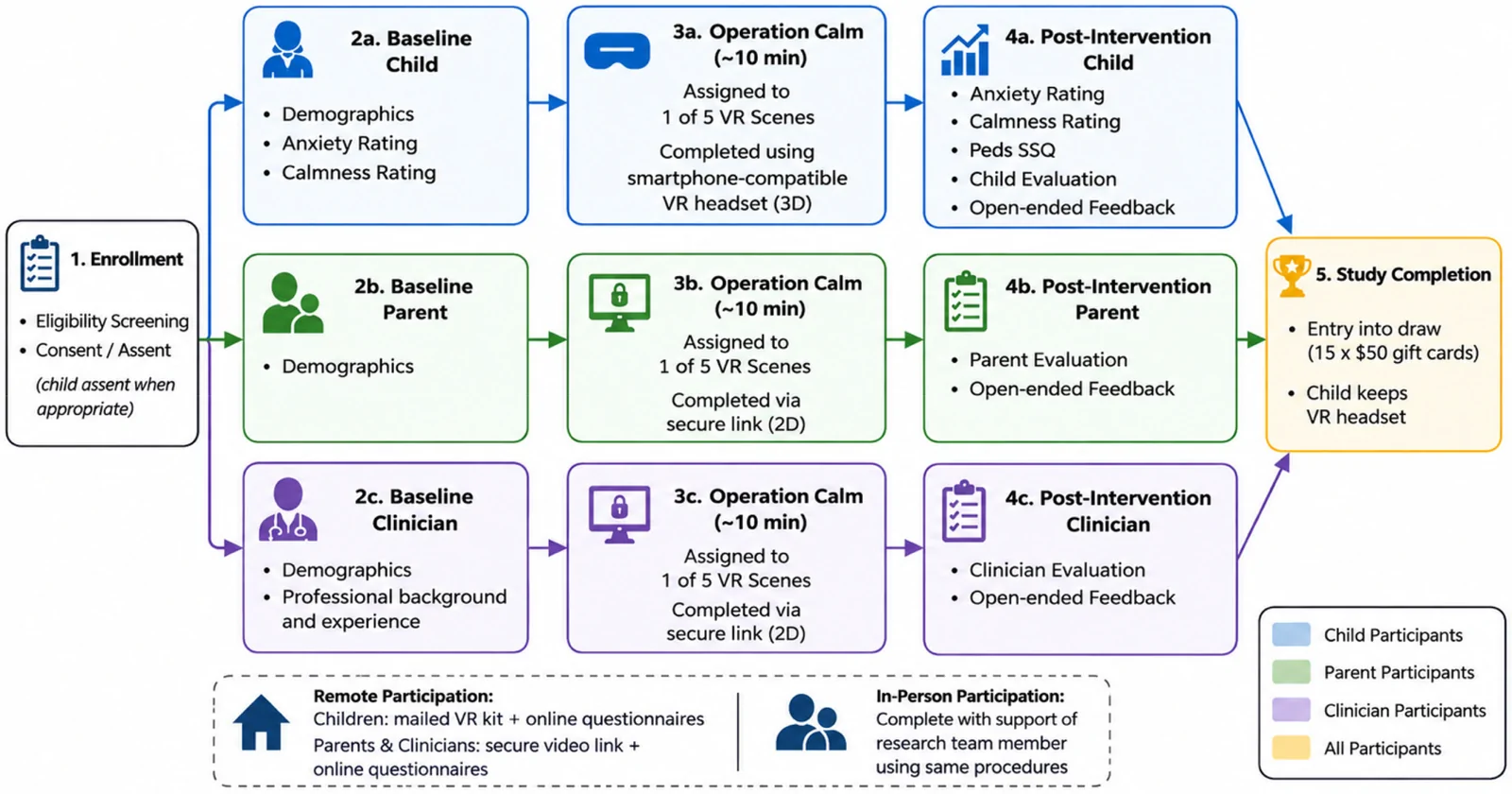
Schedule of activities.

The findings of the completed study will be reported using two complementary frameworks. The mixed-methods design, analysis, and integration of quantitative and qualitative findings will be reported in accordance with the Good Reporting of a Mixed Methods Study (GRAMMS) recommendations. The delivery of Operation Calm, including any modifications and fidelity considerations, will be reported using the Template for Intervention Description and Replication (TIDieR) checklist. Completed GRAMMS and TIDieR checklists will be provided as supplementary materials with the publication of the study findings.

### 2.2 Setting and participation modes

Children and parents may participate remotely from private homes anywhere in Canada. Study materials, including a smartphone-compatible VR headset, are mailed to remote families. Researcher-led in-person child and family sessions are limited to private homes or mutually agreed private and safe community settings in the Greater Montreal area, Quebec. Clinicians participate remotely from Canada. Recruitment and data collection are not embedded in clinical care, and the study does not use institutional medical records, school records, membership lists, or clinical recruitment pathways.

### 2.3 Participants and eligibility

The maximum planned sample is approximately 150 participants: 50 children or adolescents, up to 50 parents or legal guardians, and 50 clinicians. Groups may participate independently. Parent participation in the parent evaluation is not required for a child to participate once the applicable permission, assent, or adolescent consent is complete.

Children and adolescents are eligible if they are 6–17 years old; can understand and communicate in English or French; are willing and able to complete a seated VR session and questionnaires with neutral assistance if needed; and have the applicable authorization. For remote participation, the child or family must have access to a compatible smartphone. Children are excluded for uncontrolled epilepsy, photosensitive seizures, another VR contraindication, severe motion sickness or significant vestibular symptoms, or an unaccommodated sensory, motor, physical, or comprehension limitation that prevents safe or meaningful headset use. Acute illness, significant headache, nausea, or dizziness leads to temporary deferral rather than permanent exclusion.

Parents must be a parent or legal guardian of a child aged 6–17, be able to participate in English or French, and consent to their own study component. Clinicians must be at least 18 years old, be able to participate in English or French, and be a regulated healthcare professional or supervised trainee with pediatric care experience. Eligible disciplines may include medicine, nursing, psychology, child life, occupational therapy, physiotherapy, social work, dentistry, and allied health. Parents and clinicians review the intervention in a web-based format and are not required to wear the child headset.

### 2.4 Recruitment, consent, and assent

Participants will be recruited through REB-approved flyers, recruitment emails, Facebook and Instagram posts, and word-of-mouth sharing of public study materials. Interested individuals initiate contact through the study email or sign-up form. Group-specific screening documents age, language, participant-group requirements, technology access where applicable, and the minimum VR safety information necessary to determine eligibility. Recruitment materials state that participation is voluntary and unrelated to healthcare, school, employment, professional standing, or access to services.

Children aged 6–13 require written parental or legal-guardian permission and active child assent. Verbal, behavioural, or non-verbal dissent overrides prior assent. Adolescents aged 14–17 provide written consent after demonstrating understanding of the purpose, procedures, foreseeable risks, voluntariness, and right to withdraw, in accordance with the REB-approved process. Parents and clinicians provide separate adult consent for their components. Consent may be documented on paper in person or electronically through McGill REDCap for remote participation.

### 2.5 Scene allocation

One of five scenes is assigned using a predetermined rotating schedule intended to maintain approximately equal representation. A participating parent reviews the same scene as their child; independently participating parents and clinicians receive the next available scene. This allocation is not randomization and does not create treatment arms. Allocation may be adjusted to correct substantial imbalance and is documented in the tracking log. Scene identity will be retained in the analytic dataset so that completion, acceptability, tolerability, and exploratory outcomes can be described by scene without confirmatory between-scene testing.

### 2.6 Operation Calm intervention

Operation Calm is a self-guided nature-based VR mindfulness activity consisting of approximately 10 min of standardized, age-appropriate narration paired with a prerecorded 360° nature video. Five nature-based environments are available, including forest, beach, underwater, and wildlife settings. The mindfulness content, narration, and approximate duration are held constant while the visual environment varies. Guided elements include diaphragmatic or slow breathing, sensory grounding, present-moment awareness, gentle redirection after distraction, and brief reflection on calm, safety, and relaxation. The activity is not represented as psychotherapy, diagnosis, medical advice, or a replacement for care.

The approximately 10-minute duration was selected to balance meaningful exposure to all intervention components with feasibility, developmental attention, and tolerability in an early-stage pediatric study. The protocol tests one standardized exposure rather than attempting to establish a dose-response relationship. Longer or repeated sessions may be examined after the current study identifies acceptable content, delivery conditions, and symptom patterns.

Children use a study-supplied smartphone-compatible VR headset, as depicted in Figure 2, with a compatible family smartphone for remote participation, internet access, and the YouTube application or a standard browser to stream the prerecorded 360° video. Written setup, hygiene, supervision, and troubleshooting instructions are provided. Parents and clinicians review the assigned scene through the approved web-based format rather than the headset. Headphones are not required in the current standardized procedure; participants must be able to hear the narration clearly. Future testing may compare headphones or noise reduction where clinical environmental distraction is relevant.

**Figure 2:**
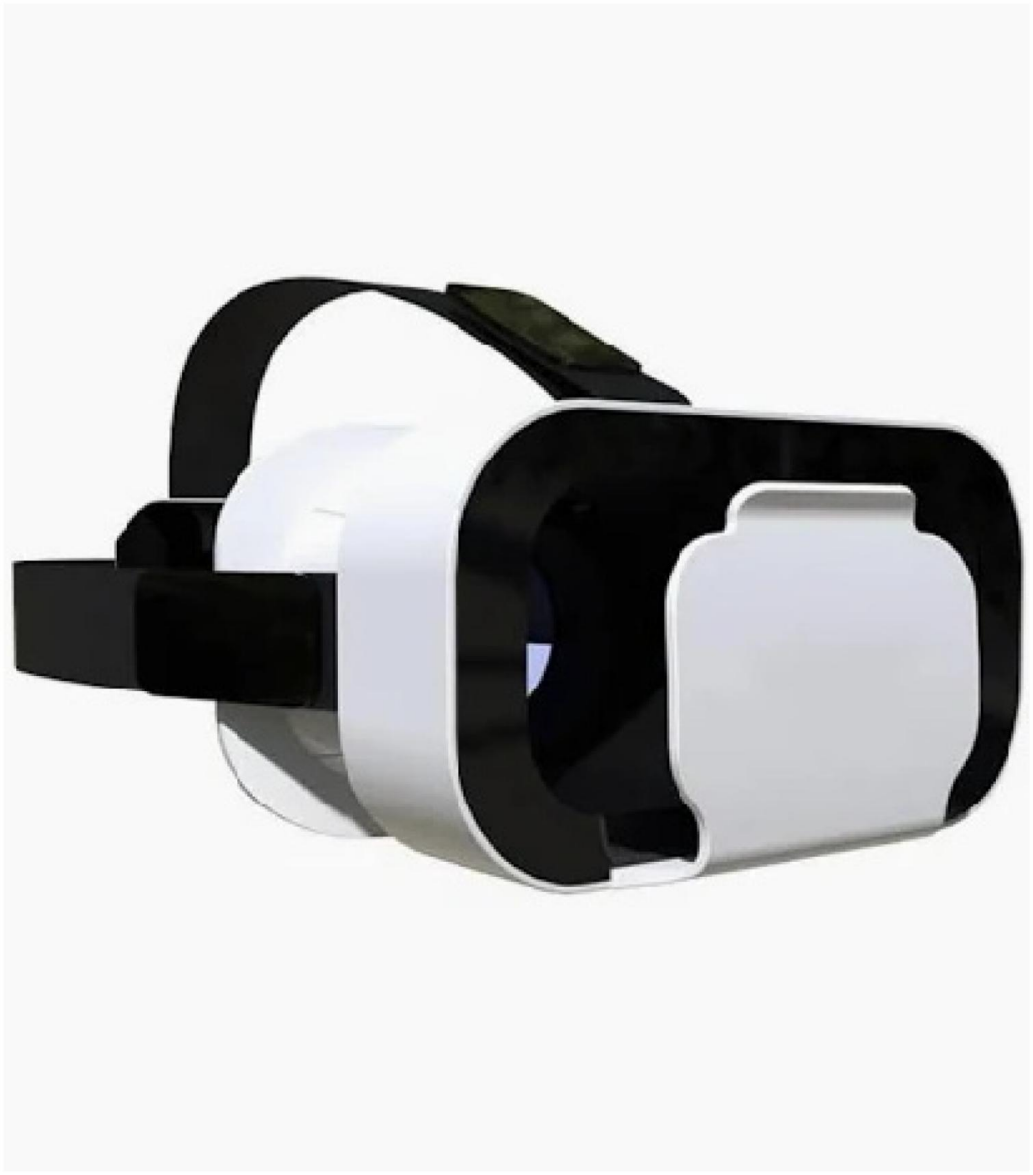
VR headset.

Intervention videos can be found here: https://www.youtube.com/playlist?list=PLGeQJg5TnB-U

### 2.7 Child procedure

• Confirm identity, eligibility, current symptoms, and completion of the correct consent/assent process; assign a participant code.
• Complete demographics with neutral reading or recording assistance when needed.
• Immediately before VR, complete the Children's Anxiety Meter–State (CAM-S) and current calmness rating.
• Review setup and safety instructions. The child remains seated in a stable supported position in a clear area; for remote sessions, a parent or legal guardian is present or readily available.
• View the assigned approximately 10-minute Operation Calm scene while listening to the standardized narration. The child may pause, remove the headset, or stop without explanation.
• Remove the headset slowly and reorient while seated. If symptoms occur, stop immediately and do not resume while symptoms continue.
• Immediately complete the post-intervention CAM-S and calmness rating, followed by the Pediatric Simulator Sickness Questionnaire (Peds SSQ) and Operation Calm Child Evaluation.
• Record scene, participation mode, timing, completion, missingness, technical problems, deviations, adverse events, and draw eligibility in the tracking log.

### 2.8 Parent and clinician procedures

After separate consent, parents complete demographics, review the same scene as their participating child or the next allocated scene if participating independently, and complete the Operation Calm Parent Evaluation. Clinicians consent electronically, complete professional demographics, review the allocated scene through the secure study link, and complete the Operation Calm Clinician Evaluation. Parent evaluations address developmental appropriateness, perceived usefulness, family practicality, safety, and clinical value. Clinician evaluations address clinical relevance, developmental fit, safety, intended settings, barriers and facilitators, resource needs, and implementation feasibility. Open-ended recommendations are collected within both forms.

### 2.9 Measures and outcomes

Feasibility is assessed through the recruitment checklist and study tracking log, including inquiries, recruitment source, eligibility, consent, enrolment, completion, timing, reminders, missingness, withdrawals, technical issues, protocol deviations, scene allocation, shipping, and adverse events. Acceptability, usability, engagement, appropriateness, perceived usefulness, and willingness to reuse or recommend are assessed through group-specific Operation Calm evaluations. Exact item wording and response options are governed by the final REB-approved English and French instruments.

The CAM-S is a brief, developmentally appropriate rating of current state anxiety administered immediately before and after VR. A separate visual and numerical current calmness rating is administered at the same time points. These immediate ratings are exploratory outcomes and are not measures of clinical treatment efficacy. Tolerability is assessed immediately after VR using the Peds SSQ and through early stops, symptom reports, and adverse-event documentation. The Peds SSQ assesses pediatric VR-related symptoms such as nausea, dizziness, headache, eye strain, fatigue, and discomfort.

### 2.10 Sample size

The target of 50 children allows for withdrawal, incomplete data, technical failure, and approximate representation of 10 children per scene. Approximately 34 children with paired observations would provide 80% power at two-sided *α* = .05 to detect a medium within-person effect (d = 0.50); however, the pre–post analysis is exploratory and is not the primary justification for the study. Parent and clinician targets are purposive and descriptive, intended to capture varied perspectives rather than power confirmatory comparisons. Final group sizes may differ because participation is independent.

### 2.11 Safety monitoring and adverse events

The study is considered minimal risk, subject to REB determination. Potential child risks include transient dizziness, nausea, headache, eye strain, blurred vision, sweating, fatigue, disorientation, balance difficulty, motion sickness, emotional discomfort, and inconvenience. Risk is reduced through contraindication screening, a single approximately 10-minute exposure, seated use in a clear area, adult availability, explicit stopping instructions, slow reorientation, symptom measurement, and adverse-event documentation. Parents and clinicians are exposed only to questionnaires and web-based review.

A session stops if the participant requests or signals stopping; moderate or severe nausea, dizziness, headache, visual disturbance, loss of balance, panic, marked distress, or unacceptable symptoms occur; continuation is judged unsafe; or equipment, environment, or supervision becomes unsuitable. The headset is removed immediately, and the child rests seated until oriented. No re-challenge is required. Events are documented by code, date, scene, description, onset, severity, duration, action, outcome, and relatedness. The principal investigator reviews safety patterns regularly and after any moderate, severe, or unexpected event. Recruitment may pause after an unexpected serious related event, repeated moderate or severe symptoms, a material increase in risk, or REB direction. An independent data safety monitoring board is not planned because exposure is brief and risk is minimal.

## 3 Data analysis

A statistical analysis file will prespecify variable names, scoring, derived outcomes, exclusions, and outlier handling before inferential analyses. Children, parents, and clinicians will first be analyzed separately. Continuous variables will be summarized using n, mean, standard deviation, median, interquartile range, minimum, and maximum as appropriate. Categorical variables will be summarized with counts, percentages, and explicit denominators. Recruitment, eligibility, consent, completion, missingness, technical-issue, early-stop, and adverse-event rates will be reported with 95% confidence intervals where informative.

Evaluation items will be summarized by group. Scale or domain scores will be calculated only when supported by the final instrument and scoring plan; otherwise items will remain descriptive. Peds SSQ items and any prespecified scores will be summarized according to approved scoring instructions. Symptoms and adverse events will be described by scene, mode, severity, relatedness, duration, and resolution while protecting confidentiality. Scene-level summaries may be displayed, but the study is not powered for definitive between-scene comparisons.

Among children with paired observations, pre–post CAM-S and calmness scores will be examined using paired-samples t tests when assumptions are reasonable or Wilcoxon signed-rank tests otherwise. Mean or median change, 95% confidence intervals, and effect sizes (paired Cohen d or r) will be reported. Two-sided *p* < .05 may be reported for these explicitly exploratory analyses; multiplicity will not be controlled, and efficacy will not be inferred. No formal multiple imputation is planned for primary descriptive outcomes. Available-case denominators and reasons for missingness, when known, will be reported. Complete pairs will be used for pre–post analyses, with sensitivity analyses considered only if missingness is substantial and assumptions are defensible.

### 3.1 Qualitative and mixed-methods analysis

Open-ended responses will undergo qualitative content analysis separately for children, parents, and clinicians. Two trained team members will independently review responses, develop and refine a coding framework, compare coding, and resolve discrepancies through discussion. Codes will be organized into categories or themes addressing strengths, limitations, usability, developmental fit, safety, barriers, facilitators, and recommended changes. An audit trail will document analytic decisions, and carefully de-identified quotations may illustrate findings.

Quantitative and qualitative findings will be integrated during interpretation using a convergent approach. Joint displays or structured matrices will compare ratings with explanatory comments and identify convergence, complementarity, or divergence. Descriptive scene profiles will synthesize completion, acceptability, engagement, usability, tolerability, exploratory anxiety and calmness changes, implementation ratings, and qualitative feedback. Refinement decisions will consider the total pattern of evidence rather than any single score. Planning benchmarks for feasibility and progression are presented in Table 1.

**Table 1:** Feasibility benchmarks.

Domain	Planning benchmark and interpretation
Child completion	≥80% supports feasibility; 60–79% suggests modification; <60% suggests major redesign.
Questionnaire completeness	≥85% with usable primary evaluation data is desirable; item-level missingness >10% triggers review.
Technical feasibility	≥80% of sessions completed without a problem preventing core procedures is desirable.
Tolerability	Any serious related event prompts pause and review; repeated moderate/severe events trigger safety modification.
Acceptability	No single cutoff; broadly favourable ratings without recurring major concern support progression.
Implementation	Clinician ratings and barrier themes define staffing, equipment, training, setting, and workflow needs.

These benchmarks are planning guides rather than efficacy thresholds. Final interpretation will consider confidence intervals, context, qualitative feedback, and any approved amendments.

### 3.2 Ethics, compensation, and registration

The study will begin only after approval by the McGill University REB and will be conducted in accordance with the Tri-Council Policy Statement: Ethical Conduct for Research Involving Humans (TCPS 2), institutional policies, and applicable Canadian and Quebec privacy requirements. Participation is voluntary, and participants may stop or withdraw without penalty. The protocol number is 26-08-078.

Children may retain the smartphone-compatible headset supplied for participation. Participants who complete their group-specific component receive one entry in the corresponding optional draw. Fifteen $50 CAD Amazon gift cards are available: five for children or adolescents, five for parents, and five for clinicians. Child prizes are issued to the parent or legal guardian. Draw contact information is stored separately from research responses, and entries are not influenced by responses or favourability of feedback.

## 4 Discussion, ethics and dissemination

This study is designed to establish whether Operation Calm is ready for subsequent clinical testing. Its contribution is not an estimate of treatment efficacy but a multi-perspective assessment of whether children can access and complete the intervention, whether they tolerate the VR exposure, whether families regard it as useful and developmentally appropriate, and whether clinicians consider it relevant and implementable. The inclusion of children, parents, and clinicians recognizes that implementation depends on more than the child's immediate experience: setup, supervision, family burden, workflow, safety, and professional confidence will influence future uptake.

The community and remote design broadens access beyond a single specialized hospital and permits evaluation across Canada, while in-person testing remains available in Greater Montreal. This design may improve the diversity of usability feedback and reveal technical challenges that would be hidden in a controlled laboratory or hospital environment. The rotating allocation of five scenes permits descriptive evaluation of content preferences and scene-specific issues while holding narration and approximate duration constant. Recording scene and delivery mode will make this heterogeneity transparent rather than treating the intervention as a uniform black box.

The approximately 10-minute standardized session is intentionally brief. At this stage, the key question is whether children can complete all intervention components comfortably and consistently. Although longer immersive exposures may be tolerated in other contexts, tolerability depends on age, hardware, movement within the content, prior VR experience, and setting. A longer session could also increase burden and confound scene acceptability with exposure duration. Findings from the Peds SSQ, early stops, and qualitative feedback can support later studies of repeated practice or longer doses.

### 4.1 Limitations

Several limitations are inherent to this early-stage design. There is no control group, randomization, allocation concealment, or blinding, and immediate change could reflect expectancy, novelty, regression to the mean, demand characteristics, or measurement reactivity. Most outcomes are self-reported, and the intervention developer's role may increase perceived pressure to provide favourable feedback despite neutral procedures. The community sample is not recruited during an actual medical or dental procedure and may have low baseline anxiety, limiting both the magnitude and clinical meaning of pre–post change. Children without compatible smartphones may be underrepresented in remote participation, and recruitment through public materials may favour families and clinicians who are interested in VR or mindfulness.

The single-session design does not address persistence, repeated use, adherence over time, or long-term transfer of coping skills. Although national remote recruitment improves geographic reach, the sample is not intended to represent all Canadian children or clinical settings. Parent and clinician web-based review is less immersive than the child headset experience, so their judgments may not fully reflect the sensory properties of VR. Scene comparisons are underpowered and confounded by non-random rotating allocation. Hardware and home internet variability may affect immersion and comfort, although these issues are themselves relevant feasibility outcomes.

### 4.2 Future research

If feasibility, acceptability, and tolerability are adequate, the next stage should evaluate Operation Calm in children facing a defined medical or dental procedure using a multicentre design, an appropriate comparator, concealed randomization where feasible, standardized co-interventions, independent outcome collection, and longer follow-up. Future trials should consider objective or observer-rated indicators such as heart rate, heart-rate variability, electrodermal activity, salivary cortisol, behavioural distress, procedural cooperation, analgesic use, and recovery outcomes to complement self-report. Studies could also examine headset type, headphones or noise cancellation, scene preference, repeated practice, dose, age-specific adaptations, French-language equivalence, accessibility, and cost or workflow outcomes.

### 4.3 Conclusion

Operation Calm combines guided mindfulness, immersive virtual nature, and a portable delivery format intended to support children's self-regulation around future healthcare stress. This mixed-methods feasibility study will determine whether the intervention can be delivered safely and consistently, how it is experienced by children and parents, and whether clinicians regard it as relevant and implementable. The findings will guide intervention refinement and the design of a future clinical feasibility study or randomized trial; they will not establish clinical efficacy.

### 4.4 Dissemination

Results may contribute to a doctoral dissertation and will be disseminated through peer-reviewed publication, academic conferences, research seminars, community or partner summaries, professional presentations, and approved institutional or study websites. Families and clinicians may be offered a plain-language summary through approved contact procedures. Only aggregate statistics and carefully de-identified quotations will be reported.
